# Supplementary material for: Metabolic activity controls the emergence of coherent flows in microbial suspensions
Source: Proc Natl Acad Sci U S A. 2025 Jan 23;122(4):e2413340122. doi: 10.1073/pnas.2413340122 (PMC11789023; doi:10.1073/pnas.2413340122)
Supplement: Supplementary file 1 — Appendix 01 (PDF) [file pnas.2413340122.sapp.pdf]

PNAS

Supporting Information for

Metabolic activity controls the emergence of coherent flows in microbial suspensions

Alexandros A. Fragkopoulos, Florian Böhme, Nicole Drewes, Oliver Bäumchen

Oliver Bäumchen.
E-mail: oliver.baeumchen@uni-bayreuth.de

This PDF file includes:

Figs. S1 to S5
Legends for Movies S1 to S2

Other supporting materials for this manuscript include the following:

Movies S1 to S2

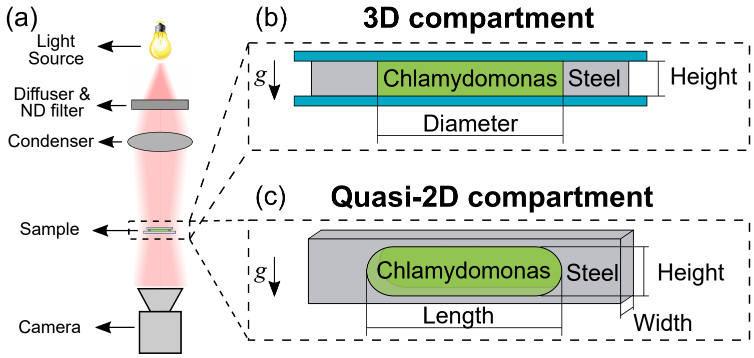

Fig. S1. Experimental setup. (**a**) A suspension containing planktonic *C. reinhardtii* cells is confined and illuminated with red light ($\lambda = 660 \pm 10$ nm) of controlled light intensities. A diffuser is used to homogenize the light, and a condenser lens focuses the light beam on the sample. The light intensity is controlled via the intensity of the light source in combination with a neutral-density filter, with OD = 1 or OD = 2. The suspension is confined in two types of compartments: (**b**) 3D and (**c**) quasi-2D compartments. (**b**) 3D compartments are cylindrical in shape with a diameter of 30 mm, while the height varies between $0.5 - 1.0$ mm. (**c**) Quasi-2D compartments have a length of 30 mm and a height of 2 mm. The width is fixed at 2 mm allowing for only one wavelength to fit. For both compartments, gravity is always along the direction of the height.

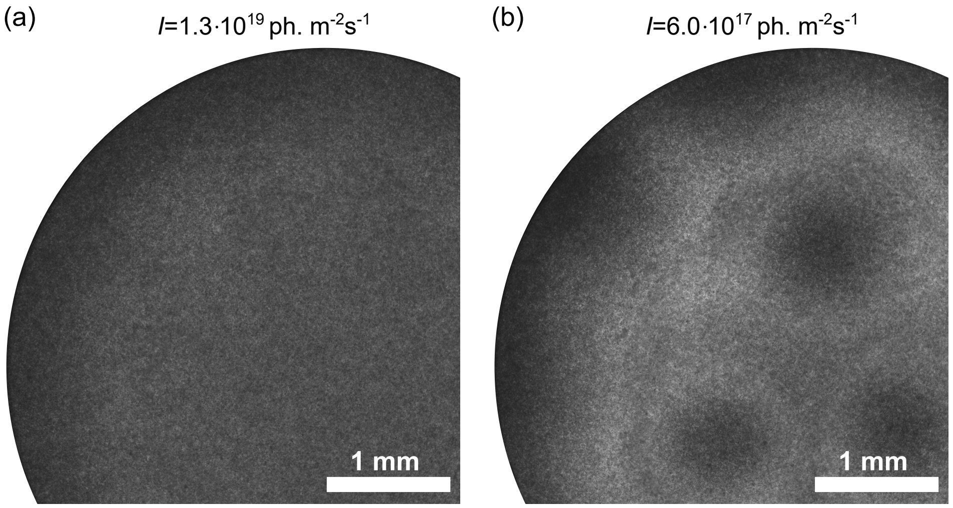

Fig. S2. Reversible bioconvection of blind mutants. A suspension of double-knockout mutants of channerlrhodopsin-1 and -2 that does not exhibit phototaxis. These blind mutants also exhibit a transition from (**a**) a homogeneous state to (**b**) a bioconvective pattern with a decreasing light intensity. These experiments were performed in disc compartments, as shown in Fig. 1b, with 8 mm diameter. The mutant strain is CC-5679 with the SAG 11-32b strain as the background strain, and was provided by the lab of Peter Hegemann at the Humboldt University of Berlin. The cells were cultivated with the same procedure as described in the methods of the main text.

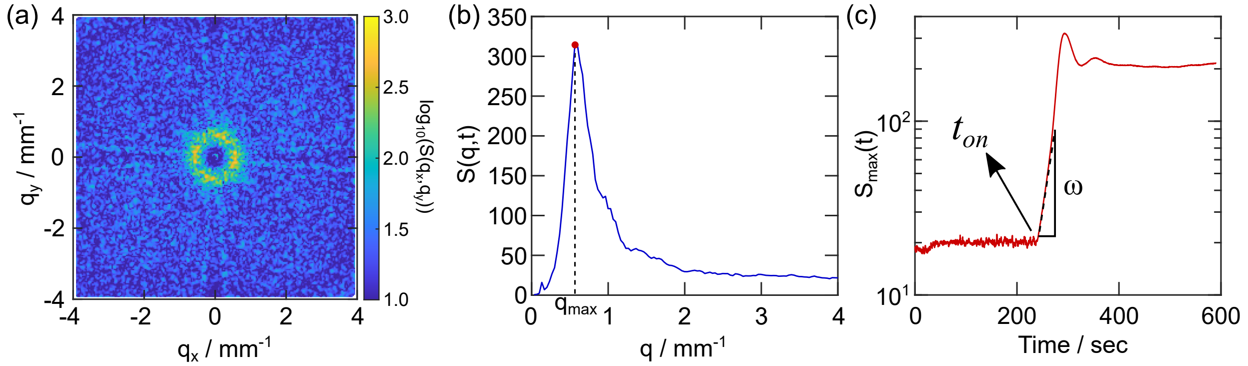

Fig. S3. Fourier analysis. (**a**) The power spectrum, $S(q_x, q_y, t)$, for an experiment in a 3D compartment with cell concentration $\rho_0 = 8 \cdot 10^7$ cells mL^{-1}, light intensity $I = 5 \cdot 10^{16}$ photons m^{-2} and 1 mm height. The spectrum was obtained after the formation of the convective pattern, i.e. 297 s after the light intensity was lowered. (**b**) The power spectrum, $S(q, t)$, was calculated using the azimuthal average of $S(q_x, q_y, t)$, corresponding to the experiment in panel (**a**). The wavenumber, $q_{\max}$, at the maximum of $S(q, t)$ indicates the wavelength of the instability, $\lambda = q_{\max}^{-1}$. (**c**) The maximum value of the power spectrum as a function of time, $S_{\max}(t)$. Using this representation, we can measure the onset time, t_{on}, and the growth rate, ω, of the instability.

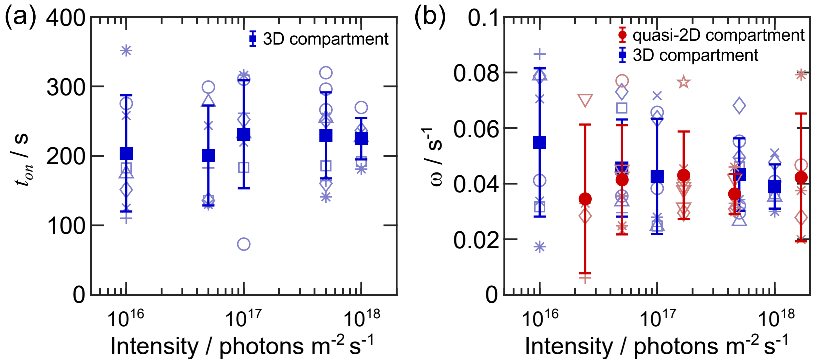

Fig. S4. Onset time and growth rates for different light intensities. (**a**) The onset time, $t_{\rm on}$, for 3D compartments is measured using the method shown in Fig. S3c. (**b**) The growth rate, ω, for both 3D and quasi-2D compartments is obtained using the method shown in Fig. S3c. Both, $t_{\rm on}$ and ω, are independent of the light intensity and the compartment type. Their mean values are $t_{on} = 220 \pm 60$ s and $\omega = (4 \pm 2) \cdot 10^{-2}$ s^{-1}. Open symbols indicate individual repetitions of biological replicates All closed symbols denote the mean and standard deviation between biological replicates.

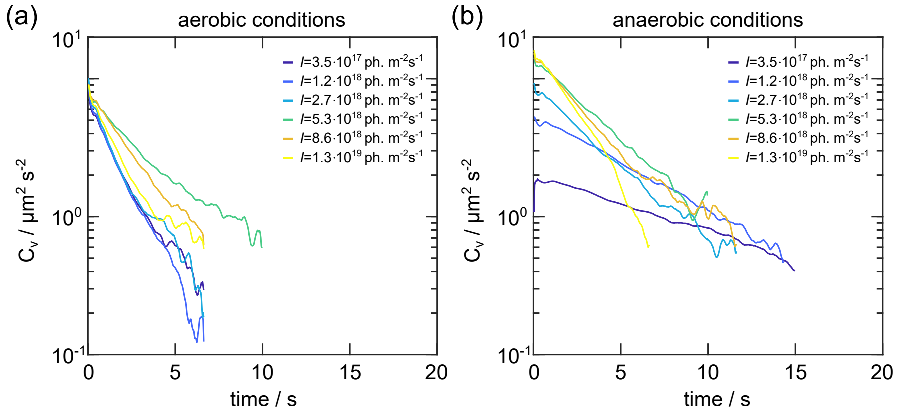

Fig. S5. Velocity autocorrelation function. The velocity autocorrelation function, C_v, is shown for suspensions in (**a**) aerobic and (**b**) anaerobic conditions. Each panel includes data from the same biological replicate for the same compartments, but at different light intensities. The order of light intensities was randomized for all experiments. Also the cells were allowed to adjust to the new light intensity for 10 minutes before recording. In all cases, the velocity autocorrelation function decays exponentially, with the slope representing the decorrelation time, $\tau_{\rm c}$, while the extrapolation to the time $t = 0$ in this representation provides the mean-square velocity, v^2.

Movie S1. Top-view of the emergence of bioconvection in an air-tight 3D compartment with $1\,\mathrm{mm}$ height. The light intensity was lowered to $I = 1 \cdot 10^{18}$ photons m^{-2} s^{-1} right at the beginning of the image sequence.

Movie S2. Side-view of the emergence of bioconvection in an air-tight quasi-2D compartment. The light intensity was lowered to $I = 4.6 \cdot 10^{18}$ photons m^{-2} s^{-1}right at the beginning of the image sequence.
